# *N*-(3,4-Dichloro­phen­yl)-3-oxo­butanamide

**DOI:** 10.1107/S1600536809051307

**Published:** 2009-12-04

**Authors:** Mukesh M. Jotani, Jerry P. Jasinski, Bharat B. Baldaniya, Ray J. Butcher

**Affiliations:** a Bhavan’s Sheth R.A. College of Science, Khanpur, Ahmedabad, Gujarat 380 001, India; b Department of Chemistry, Keene State College, 229 Main Street, Keene, NH 03435-2001, USA; c Department of Chemistry, M.G. Science Institute, Navrangpura, Ahmedabad, Gujarat 380 009, India; d Department of Chemistry, Howard University, 525 College Street NW, Washington DC 20059, USA

## Abstract

In the title compound. C_10_H_9_Cl_2_NO_2_, the acetamide residue is twisted out of the phenyl ring plane by 25.40 (9)°. An intra­molecular C—H⋯O close contact is observed. The N atom of the butanamide unit forms an inter­molecular N—H⋯O hydrogen bond with the symmetry-related carbonyl O atom, inter­linking mol­ecules into a *C*(4) chain along [100]. Additional C—H⋯O inter­molecular inter­actions and Cl⋯Cl contacts [3.4364 (8) Å] contribute to the stability of the crystal packing.

## Related literature

For the synthesis and biological activity of the title compound, see: Lliopoulos *et al.* (1986 ▶); Grissar *et al.* (1982 ▶). For related structures, see: Whitaker (1986 ▶, 1987 ▶, 1988 ▶); Whitaker & Walker (1987 ▶); Brown & Yadav (1984 ▶); Tai *et al.* (2005 ▶); Sundar *et al.* (2005 ▶); Guo (2004 ▶); Robin *et al.* (2002 ▶). For hydrogen-bond motifs, see: Bernstein *et al.* (1995 ▶). For density functional theory (DFT), see: Becke (1988 ▶, 1993 ▶); Hehre *et al.* (1986 ▶); Lee *et al.* (1988 ▶); Schmidt & Polik (2007 ▶). For the *GAUSSIAN03* program package, see: Frisch *et al.* (2004 ▶).
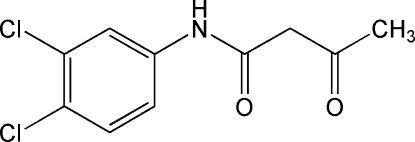


## Experimental

### 

#### Crystal data

C_10_H_9_Cl_2_NO_2_
*M* *_r_* = 246.08
Orthorhombic, 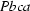

*a* = 9.7171 (4) Å
*b* = 8.2834 (5) Å
*c* = 27.4857 (16) Å
*V* = 2212.3 (2) Å^3^
*Z* = 8
Mo *K*α radiation
μ = 0.57 mm^−1^
*T* = 200 K
0.56 × 0.35 × 0.14 mm

#### Data collection

Oxford Diffraction Gemini diffractometer
Absorption correction: multi-scan (*CrysAlis RED*; Oxford Diffraction, 2007 ▶) *T*_min_ = 0.725, *T*_max_ = 0.924
16980 measured reflections
3745 independent reflections
1910 reflections with *I* > 2σ(*I*)
*R*_int_ = 0.045

#### Refinement

*R*[*F* ^2^ > 2σ(*F* ^2^)] = 0.048
*wR*(*F* ^2^) = 0.123
*S* = 1.04
3745 reflections
137 parameters
H-atom parameters constrained
Δρ_max_ = 0.21 e Å^−3^
Δρ_min_ = −0.27 e Å^−3^

### 

Data collection: *CrysAlis PRO* (Oxford Diffraction, 2007 ▶); cell refinement: *CrysAlis RED* (Oxford Diffraction, 2007 ▶); data reduction: *CrysAlis RED*; program(s) used to solve structure: *SHELXS97* (Sheldrick, 2008 ▶); program(s) used to refine structure: *SHELXL97* (Sheldrick, 2008 ▶); molecular graphics: *SHELXTL* (Sheldrick, 2008 ▶); software used to prepare material for publication: *SHELXTL* and *PLATON* (Spek, 2003 ▶).

## Supplementary Material

Crystal structure: contains datablocks global, I. DOI: 10.1107/S1600536809051307/ci2974sup1.cif

Structure factors: contains datablocks I. DOI: 10.1107/S1600536809051307/ci2974Isup2.hkl

Additional supplementary materials:  crystallographic information; 3D view; checkCIF report

## Figures and Tables

**Table 1:** Hydrogen-bond geometry (Å, °)

*D*—H⋯*A*	*D*—H	H⋯*A*	*D*⋯*A*	*D*—H⋯*A*
N—H0*A*⋯O1^i^	0.88	1.95	2.824 (2)	172
C6—H6*A*⋯O1	0.95	2.35	2.865 (2)	113
C2—H2*A*⋯O2^ii^	0.95	2.58	3.345 (2)	138
C8—H8*A*⋯O2^iii^	0.99	2.45	3.327 (2)	147

Symmetry codes: (i) 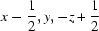; (ii) 
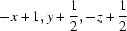
; (iii) 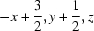.

**Fig. 1.**
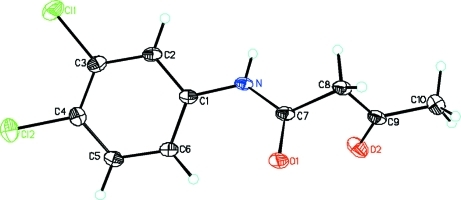
The molecular structure of C10H9NO2Cl2, (I), showing the atom-numbering scheme and 50% probability displacement ellipsoids.

**Fig. 2.**
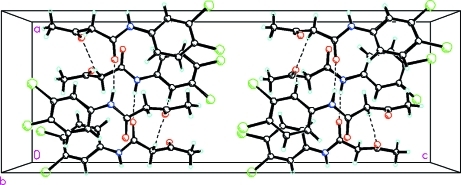
Molecular packing for (I) viewed down the b axis. Dashed lines indicate N—H···O and C—H···O intermolecular hydrogen bonds.

## supplementary crystallographic information

### Comment

Acetoacetanilide, a very useful chemical intermediate in the production of pigments (Whitaker, 1986, 1987, 1988; Whitaker & Walker, 1987; Brown & Yadav, 1984) possesses cardiotonic, antihypertensive and anti-thrombic properties (Grissar *et al.*, 1982). The title compound, (I), is used as an intermediate in the synthesis of acetoacetanilide and a variety of other biologically important heterocyclic compounds containing pyridine, pyrimidine and imidazole. In the view of the importance of (I), its crystal structure is determined.

In (I), the C═O bond lengths are 1.2292 (18) Å and 1.207 (2) Å which confirms that the compound is in the keto form (Fig. 1). The phenyl ring (C1–C6) is planar with a maximum deviation of 0.007 (1) Å for the C1 atom, from the least-squares plane of the ring. The short C—N distances of 1.407 (2) and 1.346 (2) Å and C1—N—C7 larger bond angle of 126.9 (13)° may be attributed to the involvement of the butanamide N atom in the intermolecular N—H···O interaction and a short intramolecular contact (1.95 Å) between O1 and H0A which is less than their van der Waals radii (2.72 Å). Similar short contacts are also observed in other related structures containing the acetamide residue (Sundar *et al.*, 2005; Guo, 2004; Robin *et al.*, 2002). Atoms N, C7, O1 and C8 forming the acetamide residue are coplanar with a maximum deviation of -0.005 (2) Å for the C7 atom. The acetamide residue is twisted considerably from the least-squares plane of phenyl ring having a dihedral angle of 25.40 (9)°. Atoms C8, C9, O2 and C10 from the *O*-acetyl group are also coplanar displaying a dihedral angle of 49.21 (10)° with the mean plane of the phenyl ring (C1—C6) and 73.78 (11)° with the least-squares plane of the acetamide residue.

The N atom in the butanamide moiety forms an intermolecular hydrogen bond (N—H0A···O1) with the symmetry related carbonyl oxygen atom interlinking molecules into an one-dimensional chain along the [100] (Fig. 2 and Table 1) forming a C(4) graph-set motif (Bernstein *et al.*,1995). Torsional angles C7—C8—C9—O2 (15.9 (2)°) and O1—C7—C8—C9 (67.3 (2)°) about C8—C9 and C7—C8, respectively, suggest the involvement of O1 and O2 atoms in a weak C—H···O1 intermolecular hydrogen bonding interaction. Atoms C2 from the phenyl ring (C1–C6) and C8 from the butanamide group form weak, bifurcated intermolecular hydrogen bonds with nearby symmetry related O2 atoms (Table 2). In addition, a short intramolecular C—H···O contact (Table 2) and a weak intermolecular Cl···Cl contact (3.4364 (8) Å) exists which influences crystal packing.

Following a density functional theory calculation (Schmidt & Polik 2007) at the B3LYP 6–31-G(*d*) level (Becke, 1988, 1993; Lee *et al.* 1988; Hehre *et al.* 1986) with the *GAUSSIAN03* program package (Frisch *et al.* 2004) the angle between the mean planes of the C8/C9/O2/C10 and N/C7/O1/C8 groups change from 73.7 (8)° to 33.0 (2)°. The angle between the least-squares plane of the benzene ring and the mean planes of the C8/C9/O2/C10 and N/C7O1/C8 groups change from 49.2 (1)° and 25.4 (1)° to 30.1 (5)° and 3.6 (5)°, respectively. This results in twisting the C8═O2 keto group to be in the proximity of the butanamide N atom forming a pseudo intramolecular N—H···O hydrogen bond interaction (D–H = 1.02 (0) Å; H···A = 1.92 (0) Å; D···A = 2.76 (1) Å; D–H···A = 137.6 (9)°). These results support the collective effects of the intra and intermolecular hydrogen bonding described above influencing crystal packing.

### Experimental

The title compound was prepared by a method similar to that of Lliopoulos *et al.* (1986). A solution of 3,4-dichloroaniline (10 mmol) in benzene (30 ml) was added to a solution of ethyl acetoacetate (10 mmol) and the reaction mixture was refluxed for 2 h with stirring. The resulting precipitate was collected by filtration, washed several times with benzene and dried *in vacuo* (yield 86%). An ethanol solution of the title compound was allowed to evaporate slowly and colorless crystals of (I) were obtained after a week.

### Refinement

All of the H atoms were placed in their calculated positions and then refined using the riding model with C–H = 0.95–0.99 Å, N–H = 0.88Å and with *U*_iso_(H) = 1.19–1.50*U*_eq_(C) and 1.18*U*_eq_(N).

### Crystal data

**Table:**

C_10_H_9_Cl_2_NO_2_	*F*(000) = 1008
*M**_r_* = 246.08	*D*_x_ = 1.478 Mg m^−^^3^
Orthorhombic, *P**b**c**a*	Mo *K*α radiation, λ = 0.71073 Å
Hall symbol: -P 2ac 2ab	Cell parameters from 4228 reflections
*a* = 9.7171 (4) Å	θ = 4.9–32.4°
*b* = 8.2834 (5) Å	µ = 0.56 mm^−^^1^
*c* = 27.4857 (16) Å	*T* = 200 K
*V* = 2212.3 (2) Å^3^	Plate, colorless
*Z* = 8	0.56 × 0.35 × 0.14 mm

### Data collection

**Table:**

Oxford Diffraction Gemini diffractometer	3745 independent reflections
Radiation source: fine-focus sealed tube	1910 reflections with *I* > 2σ(*I*)
graphite	*R*_int_ = 0.045
Detector resolution: 10.5081 pixels mm^-1^	θ_max_ = 32.5°, θ_min_ = 4.9°
φ and ω scans	*h* = −14→14
Absorption correction: multi-scan (*CrysAlis RED*; Oxford Diffraction, 2007)	*k* = −11→12
*T*_min_ = 0.725, *T*_max_ = 0.924	*l* = −39→39
16980 measured reflections	

### Refinement

**Table:**

Refinement on *F*^2^	Primary atom site location: structure-invariant direct methods
Least-squares matrix: full	Secondary atom site location: difference Fourier map
*R*[*F*^2^ > 2σ(*F*^2^)] = 0.048	Hydrogen site location: inferred from neighbouring sites
*wR*(*F*^2^) = 0.123	H-atom parameters constrained
*S* = 1.04	*w* = 1/[σ^2^(*F*_o_^2^) + (0.0532*P*)^2^ + 0.0634*P*] where *P* = (*F*_o_^2^ + 2*F*_c_^2^)/3
3745 reflections	(Δ/σ)_max_ = 0.001
137 parameters	Δρ_max_ = 0.21 e Å^−^^3^
0 restraints	Δρ_min_ = −0.26 e Å^−^^3^

### Special details

**Table:**

Geometry. All e.s.d.'s (except the e.s.d. in the dihedral angle between two l.s. planes) are estimated using the full covariance matrix. The cell e.s.d.'s are taken into account individually in the estimation of e.s.d.'s in distances, angles and torsion angles; correlations between e.s.d.'s in cell parameters are only used when they are defined by crystal symmetry. An approximate (isotropic) treatment of cell e.s.d.'s is used for estimating e.s.d.'s involving l.s. planes.
Refinement. Refinement of *F*^2^ against ALL reflections. The weighted *R*-factor *wR* and goodness of fit *S* are based on *F*^2^, conventional *R*-factors *R* are based on *F*, with *F* set to zero for negative *F*^2^. The threshold expression of *F*^2^ > σ(*F*^2^) is used only for calculating *R*-factors(gt) *etc*. and is not relevant to the choice of reflections for refinement. *R*-factors based on *F*^2^ are statistically about twice as large as those based on *F*, and *R*- factors based on ALL data will be even larger.

### Fractional atomic coordinates and isotropic or equivalent isotropic displacement parameters (Å^2^)

**Table:**

	*x*	*y*	*z*	*U*_iso_*/*U*_eq_	
Cl1	0.44746 (6)	0.24662 (8)	0.45002 (2)	0.0723 (2)	
Cl2	0.72279 (6)	0.05651 (7)	0.47381 (2)	0.0685 (2)	
O1	0.79285 (11)	0.25949 (19)	0.23755 (5)	0.0569 (4)	
O2	0.64604 (16)	0.07935 (16)	0.15306 (6)	0.0661 (4)	
N	0.58187 (12)	0.24207 (16)	0.27204 (5)	0.0362 (3)	
H0A	0.4932	0.2505	0.2661	0.043*	
C1	0.61935 (15)	0.19559 (18)	0.31946 (6)	0.0335 (4)	
C2	0.52923 (16)	0.2333 (2)	0.35679 (7)	0.0390 (4)	
H2A	0.4453	0.2872	0.3496	0.047*	
C3	0.56032 (17)	0.1931 (2)	0.40451 (7)	0.0429 (4)	
C4	0.68175 (19)	0.1122 (2)	0.41514 (7)	0.0431 (4)	
C5	0.77092 (18)	0.0733 (2)	0.37786 (7)	0.0444 (4)	
H5A	0.8543	0.0185	0.3851	0.053*	
C6	0.74098 (17)	0.1129 (2)	0.33015 (7)	0.0401 (4)	
H6A	0.8027	0.0840	0.3048	0.048*	
C7	0.66728 (16)	0.2750 (2)	0.23478 (7)	0.0382 (4)	
C8	0.60112 (17)	0.3356 (2)	0.18869 (7)	0.0420 (4)	
H8A	0.6441	0.4395	0.1795	0.050*	
H8B	0.5023	0.3560	0.1949	0.050*	
C9	0.61455 (17)	0.2188 (2)	0.14672 (7)	0.0434 (4)	
C10	0.5849 (2)	0.2844 (3)	0.09710 (8)	0.0644 (6)	
H10A	0.5898	0.1969	0.0732	0.097*	
H10B	0.6529	0.3675	0.0890	0.097*	
H10C	0.4925	0.3319	0.0967	0.097*	

### Atomic displacement parameters (Å^2^)

**Table:**

	*U*^11^	*U*^22^	*U*^33^	*U*^12^	*U*^13^	*U*^23^
Cl1	0.0658 (4)	0.0910 (5)	0.0601 (3)	0.0116 (3)	0.0270 (3)	0.0049 (3)
Cl2	0.0810 (4)	0.0739 (4)	0.0507 (3)	−0.0003 (3)	−0.0064 (3)	0.0127 (3)
O1	0.0190 (6)	0.0920 (11)	0.0598 (8)	0.0011 (6)	0.0034 (5)	0.0184 (8)
O2	0.0785 (10)	0.0437 (8)	0.0759 (11)	0.0137 (7)	−0.0205 (8)	0.0024 (7)
N	0.0162 (5)	0.0413 (8)	0.0510 (8)	0.0006 (6)	0.0005 (6)	0.0015 (7)
C1	0.0230 (7)	0.0275 (8)	0.0500 (10)	−0.0048 (6)	−0.0003 (7)	−0.0027 (7)
C2	0.0258 (8)	0.0354 (9)	0.0557 (11)	−0.0007 (7)	0.0047 (7)	−0.0004 (8)
C3	0.0395 (10)	0.0379 (9)	0.0514 (11)	−0.0054 (8)	0.0113 (8)	0.0002 (8)
C4	0.0477 (10)	0.0373 (9)	0.0443 (11)	−0.0068 (8)	−0.0028 (8)	0.0046 (8)
C5	0.0388 (9)	0.0372 (10)	0.0572 (12)	0.0056 (8)	−0.0034 (9)	0.0019 (9)
C6	0.0314 (8)	0.0382 (10)	0.0508 (10)	0.0051 (7)	0.0012 (8)	−0.0022 (8)
C7	0.0232 (7)	0.0385 (9)	0.0530 (10)	−0.0001 (7)	0.0013 (7)	0.0031 (8)
C8	0.0285 (8)	0.0382 (10)	0.0593 (12)	0.0045 (8)	0.0020 (8)	0.0094 (8)
C9	0.0276 (8)	0.0425 (11)	0.0601 (12)	0.0027 (8)	−0.0050 (8)	0.0089 (9)
C10	0.0637 (13)	0.0739 (15)	0.0558 (13)	0.0119 (11)	−0.0047 (11)	0.0150 (11)

### Geometric parameters (Å, °)

**Table:**

Cl1—C3	1.7215 (18)	C4—C5	1.380 (3)
Cl2—C4	1.7241 (19)	C5—C6	1.383 (3)
O1—C7	1.2292 (18)	C5—H5A	0.95
O2—C9	1.207 (2)	C6—H6A	0.95
N—C7	1.346 (2)	C7—C8	1.507 (2)
N—C1	1.407 (2)	C8—C9	1.511 (3)
N—H0A	0.88	C8—H8A	0.99
C1—C2	1.385 (2)	C8—H8B	0.99
C1—C6	1.397 (2)	C9—C10	1.496 (3)
C2—C3	1.387 (3)	C10—H10A	0.98
C2—H2A	0.95	C10—H10B	0.98
C3—C4	1.388 (3)	C10—H10C	0.98
			
C7—N—C1	126.91 (13)	C1—C6—H6A	120.2
C7—N—H0A	116.5	O1—C7—N	122.94 (16)
C1—N—H0A	116.5	O1—C7—C8	120.69 (16)
C2—C1—C6	119.32 (16)	N—C7—C8	116.37 (13)
C2—C1—N	117.45 (14)	C7—C8—C9	113.05 (15)
C6—C1—N	123.22 (15)	C7—C8—H8A	109.0
C1—C2—C3	120.60 (15)	C9—C8—H8A	109.0
C1—C2—H2A	119.7	C7—C8—H8B	109.0
C3—C2—H2A	119.7	C9—C8—H8B	109.0
C2—C3—C4	120.01 (16)	H8A—C8—H8B	107.8
C2—C3—Cl1	119.13 (13)	O2—C9—C10	121.89 (19)
C4—C3—Cl1	120.86 (15)	O2—C9—C8	121.61 (18)
C5—C4—C3	119.36 (17)	C10—C9—C8	116.50 (17)
C5—C4—Cl2	119.13 (14)	C9—C10—H10A	109.5
C3—C4—Cl2	121.51 (15)	C9—C10—H10B	109.5
C4—C5—C6	121.12 (16)	H10A—C10—H10B	109.5
C4—C5—H5A	119.4	C9—C10—H10C	109.5
C6—C5—H5A	119.4	H10A—C10—H10C	109.5
C5—C6—C1	119.57 (16)	H10B—C10—H10C	109.5
C5—C6—H6A	120.2		
			
C7—N—C1—C2	152.83 (15)	Cl2—C4—C5—C6	178.76 (14)
C7—N—C1—C6	−27.9 (2)	C4—C5—C6—C1	0.9 (3)
C6—C1—C2—C3	1.5 (2)	C2—C1—C6—C5	−1.5 (2)
N—C1—C2—C3	−179.14 (15)	N—C1—C6—C5	179.22 (15)
C1—C2—C3—C4	−1.0 (3)	C1—N—C7—O1	3.9 (3)
C1—C2—C3—Cl1	178.40 (13)	C1—N—C7—C8	−175.10 (15)
C2—C3—C4—C5	0.3 (3)	O1—C7—C8—C9	67.3 (2)
Cl1—C3—C4—C5	−179.00 (14)	N—C7—C8—C9	−113.63 (17)
C2—C3—C4—Cl2	−178.72 (14)	C7—C8—C9—O2	15.9 (2)
Cl1—C3—C4—Cl2	1.9 (2)	C7—C8—C9—C10	−164.88 (15)
C3—C4—C5—C6	−0.3 (3)		

### Hydrogen-bond geometry (Å, °)

**Table:**

*D*—H···*A*	*D*—H	H···*A*	*D*···*A*	*D*—H···*A*
N—H0A···O1^i^	0.88	1.95	2.824 (2)	172
C6—H6A···O1	0.95	2.35	2.865 (2)	113
C2—H2A···O2^ii^	0.95	2.58	3.345 (2)	138
C8—H8A···O2^iii^	0.99	2.45	3.327 (2)	147

Symmetry codes: (i) *x*−1/2, *y*, −*z*+1/2; (ii) −*x*+1, *y*+1/2, −*z*+1/2; (iii) −*x*+3/2, *y*+1/2, *z*.

## References

[bb1] Becke, A. D. (1988). *Phys. Rev.* A**38**, 3098–100. 10.1103/physreva.38.3098 9900728

[bb2] Becke, A. D. (1993). *J. Chem. Phys.* **98**, 5648–5652.

[bb3] Bernstein, J., Davis, R. E., Shimoni, L. & Chang, N. L. (1995). *Angew. Chem. Int. Ed. Eng.* **34**, 1555–1573.

[bb4] Brown, C. J. & Yadav, H. R. (1984). *Acta Cryst.* C**40**, 564–566.

[bb5] Frisch, M. J., *et al.* (2004). *GAUSSIAN03* Gaussian Inc., Wallingford, CT, USA.

[bb6] Grissar, J. M., Schnettler, R. A. & Dage, R. C. (1982). US Patent 4329470.

[bb7] Guo, M.-L. (2004). *Acta Cryst.* E**60**, o736–o737.

[bb8] Hehre, W. J., Random, L., Schleyer, P. & Pople, J. A. (1986). *Ab Initio Molecular Orbital Theory* New York: Wiley.

[bb9] Lee, C., Yang, W. & Parr, R. G. (1988). *Phys. Rev.* **B37**, 785–789. 10.1103/physrevb.37.785 9944570

[bb10] Lliopoulos, P., Fallon, G. D. & Murray, S. (1986). *J. Chem. Soc. Dalton Trans.* pp. 437–443.

[bb11] Oxford Diffraction (2007). *CrysAlis PRO *and *CrysAlis RED* Oxford Diffraction Ltd, Abingdon, England.

[bb12] Robin, M., Galy, J.-P., Kenz, A. & Pierrot, M. (2002). *Acta Cryst.* E**58**, o644–o645.

[bb13] Schmidt, J. R. & Polik, W. F. (2007). *WebMO Pro.*WebMO, LLC: Holland, MI, USA; available from http://www.webmo.net.

[bb14] Sheldrick, G. M. (2008). *Acta Cryst.* A**64**, 112–122. 10.1107/S0108767307043930 18156677

[bb15] Spek, A. L. (2003). *J. Appl. Cryst.* **36**, 7–13.

[bb16] Sundar, T. V., Parthasarathi, V., Walfort, B., Lang, H., Piplani, P. & Malik, R. (2005). *Acta Cryst.* E**61**, o2868–o2870.

[bb17] Tai, X.-S., Liu, W.-Y., Liu, Y.-Z. & Li, Y.-Z. (2005). *Acta Cryst.* E**61**, o389–o390.

[bb18] Whitaker, A. (1986). *Acta Cryst.* C**42**, 1566–1569.

[bb19] Whitaker, A. (1987). *Acta Cryst.* C**43**, 2141–2144.

[bb20] Whitaker, A. (1988). *Acta Cryst.* C**44**, 1587–1590.

[bb21] Whitaker, A. & Walker, N. P. C. (1987). *Acta Cryst.* C**43**, 2137–2141.

